# Cytogenetic Analysis of Histone Gene and DNA Methylation in Amazonian Turtles (Chelonia: Podocnemididae)

**DOI:** 10.1021/acsomega.5c05643

**Published:** 2025-08-18

**Authors:** Ana Beatriz P. Costa-Richene, Bruno R. R. Almeida, Flávia S. Tavares, Luan F. S. Frade, André L. A. Sá, Luís A. S. Nascimento, Cleusa Y. Nagamachi, Julio C. Pieczarka, Maria I. C. Sampaio, Geraldo N. R Filho, Adauto L. Cardoso, Cesar Martins, Renata C. R. Noronha

**Affiliations:** † Genetics and Cellular Biology Laboratory, Center for Biodiversity Studies, 37871 Federal University of Pará, Belém 66075-110, Pará, Brazil; ‡ University of the State of Pará, Cametá 68400-000, Pará, Brazil; § Applied Genetics Laboratory, Socio-Environmental and Water Resources Institute, Federal Rural University of the Amazon, Belém 66077-530, Pará, Brazil; ∥ Amazon Oil Laboratory, Federal University of Pará, Belém 66075-110, Pará, Brazil; ⊥ Cytogenetics Laboratory, Center for Advanced Biodiversity Studies, Federal University of Pará, Belém 66075-110, Pará, Brazil; # Instituto de Estudos Costeiros, Federal University of Pará, Bragança 68600-971, Pará, Brazil; ∇ Integrative Genomics Laboratory, Department of Structural and Functional Biology, Botucatu Biosciences Institute, Universidade Estadual Paulista, Botucatu 18618-687, São Paulo, Brazil

## Abstract

Histone genes contain sequences responsible for coding five types of proteins (H1, H2A, H2B, H3, and H4) that are of great importance for chromatin organization. Their transcriptional regulation through DNA methylation has been little studied. Testudines are ancient reptiles with high cytogenetic diversity (2*n* = 26–68), with a large number of histone gene loci in their karyotype. The aim of this study was to analyze the physical location of the histone genes H2A, H2B, and H4 and the regions rich in 5-methylcytosine (5mC) and 5-hydroxy-methylcytosine (5hmC) in the karyotypes of *Podocnemis expansa* and *Podocnemis unifilis*. The results showed that *P. expansa* and *P. unifilis* have a diploid number of 2*n* = 28, NF = 50 and 46, respectively. Fluorescence *in situ* hybridization (FISH) showed clusters of histone genes in both species, with histone H2A, H2B, and H4 sequences located in the pericentromeric region of several pairs of karyotypes. Interstitial clusters of histone H2A and histone H4 genes were observed in the chromosome arms of *P. expansa* and *P. unifilis*. Immunolocalization of 5mC in *P. unifilis* and *P. expansa* showed that highly methylated regions are distributed mainly in the centromeres, coinciding with many loci H2A, H2B, and H4 genes, as well as markings observed along chromosome arms and distal regions. In relation to 5hmc, it was observed that most of this epigenetic mark has a dispersed distribution along the chromatids and in the distal regions in *P. unifilis* and *P. expansa*. Our findings suggest that ectopic recombination and inversion-type rearrangements may contribute to the dispersion of histone genes in *Podocnemis* and that DNA hypermethylation may be an important mechanism for the inactivation of these multigens during the cell cycle in the turtles studied.

## Graphical abstract

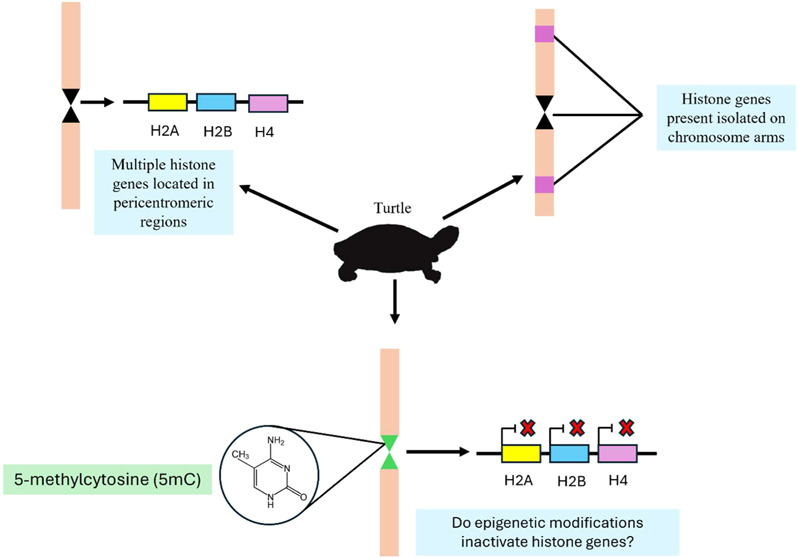

## Introduction

One of the objectives of cytogenetic study is the physical mapping of repetitive DNAs, as this reveals important information about the structure and evolution of the genome, as well as allowing the detection of structural chromosomal alterations.^1^ They comprise sequences that occur in multiple copies in the eukaryotic genome, such as multigene families (rDNA, histone genes, U snDNA, etc.) and transposable elements.^2^ Histone (DNA-hist) genes contain sequences responsible for encoding histone H1, H2A, H2B, H3, and H4 proteins, which are involved in important cellular roles such as transcription regulation, heterochromatin formation, and DNA repair.^3^ These genes differ from other eukaryotic coding sequences because (1) they do not have introns; (2) they generate an mRNA without the poly-A tail; and (3) they are associated with a nuclear body that contains proteins that act in the processing of DNA-hist mRNAs, the HLB (histone locus body).^4^ Although they are considered to be quite conserved, their organization *in situ* on chromosomes can vary between different groups of organisms, presenting one or multiple clusters, or even dispersed in small copies throughout the genome.^5^ The physical mapping of histone genes can provide insights into the mechanisms that act in their diversification, evolution, and chromosomal function,^6,7^ so they are excellent markers for inferring rearrangements involved in karyotypic evolution and the origin of sex and supernumerary chromosomes.^8^

The order Testudines includes turtles and tortoises. This group of reptiles is considered to be the oldest in existence, with the first fossil evidence dating back to the Permian period approximately 280 million years ago and are unique among tetrapods in possessing a bony shell that is integrated into the axial skeleton.^9^ There are two suborders in this order: Cryptodira and Pleurodira (Chelidae, Pelomedusidae, and Podocnemidae). The Podocnemidae family corresponds to a monophyletic group, represented by chelonians classified into 3 genera: *Erymnochelys*, *Peltocephalus*, and *Podocnemis*.^10^ The *Podocnemis* genus is widely distributed throughout South America and is represented by species *P. vogli*, *P. lewyana*, *P. erythrocephala*, *P. expansa* (PEX), *P. sextuberculata*, and *P. unifilis* (PUN), but only the last four occur in Brazil.^10^ Chelonians are long-lived, with slow sexual maturation, which results in a low rate of replacement of individuals, as well as being important seed dispersers, contributing to ecological balance.^11^

Despite the great diversity of species in the Podocnemididae family and their high importance for the fauna of the Amazon region, cytogenetic studies by this group are limited. Cytogenetic analyses have contributed to the taxonomic classification of chelonians as they provide important data to help delimit species and infer the chromosomal evolution that has occurred in these taxa.^12^ Karyotype diversity in the order Testudines is high, with diploid numbers (2*n*) ranging from 26 to 68 chromosomes, dividing the order into three distinct groups: (I) karyotypes with high diploid numbers (2*n* = 60–68 chromosomes); (II) karyotypes with intermediate diploid numbers (2*n* = 50–56); and (III) karyotypes with low diploid numbers (2*n* = 26–28), which include members of the Podocnemidae family.^13^ In the Podocnemidae family, only data on the *in situ* chromosomal location of histone H1 and H3 genes are known for members of the genera *Podocnemis* ^12^ and *Rhinoclemmys*.^14^ In both cases, a large number of these DNA-hist clusters were observed in the PEX and PUN karyotypes.

In addition, epigenetic analyses are an important tool for understanding the mechanisms of gene expression regulation and how these mechanisms are influenced by the environment and inheritance.^15^ Epigenetic analysis has been applied in different lines of research, such as cancer,^16^ neurodegenerative disorders,^17^ and epigenomics in plants^18^ and has been gaining prominence in the chelonian group, showing increasingly promising ways of understanding gene regulation in various environmental influences that these organisms are subject to. An important epigenetic mechanism of transcriptional silencing is DNA methylation, which consists of the addition of a methyl radical (CH3) to carbon 5 of cytosine, giving rise to 5-methylcytosine, mainly in gene promoter regions.^19^ This addition is made by DNA methyltransferase enzymes (DNMTs) and can be undone, a process called demethylation.^20^ In addition, another process that can occur is the oxidation of 5mC, a reaction catalyzed by TET (ten–eleven translocation) enzymes, which adds the hydroxymethyl group (−CH2OH) by binding to carbon 5 of cytosine, forming 5-hydroxymethylcytosine (5hmC).^21^ DNA methylation contributes to genome stability and plays an important role in genomic imprinting.^22^ These epigenetic alterations can act on processes at the chromosomal level, as in the case of the inactivation of sex chromosomes.^23^

Considering the importance of cytogenetic analyses of *in situ* distribution patterns of histone sequences in the karyotype of Podocnemidae, this study aims to comparatively analyze the physical location of the histone genes H2A, H2B, and H4 and the regions rich in 5-methylcytosine (5mC) and 5-hydroxymethylcytosine (5hmC) in the PUN and PEX karyotypes in order to contribute to understanding the epigenetic regulation of these multigenic families and propose hypotheses about their evolutionary genomic dynamics.

## Materials and Methods

### Obtaining the Sample

Approximately 10 mL of blood was collected from the tail vein of six PEX samples and four PUN samples from the municipality of Capitão-Poço (1°44′47″ S, 47°3′57″ W), state of Pará, Brazil (Figure 1), and kept in EDTA tubes. Additionally, a piece of tissue each from a male and a female PEX and PUN was preserved in 100% ethanol. The studies were conducted in strict compliance with ethical recommendations for the use and management of chelonians in research, under a protocol approved by the Ethics Committee for Research with Experimental Animals (Protocol 8803211223) and by the Biodiversity Authorization and Information System (SISBIO; License Number 42642-5).

**Figure 1:**
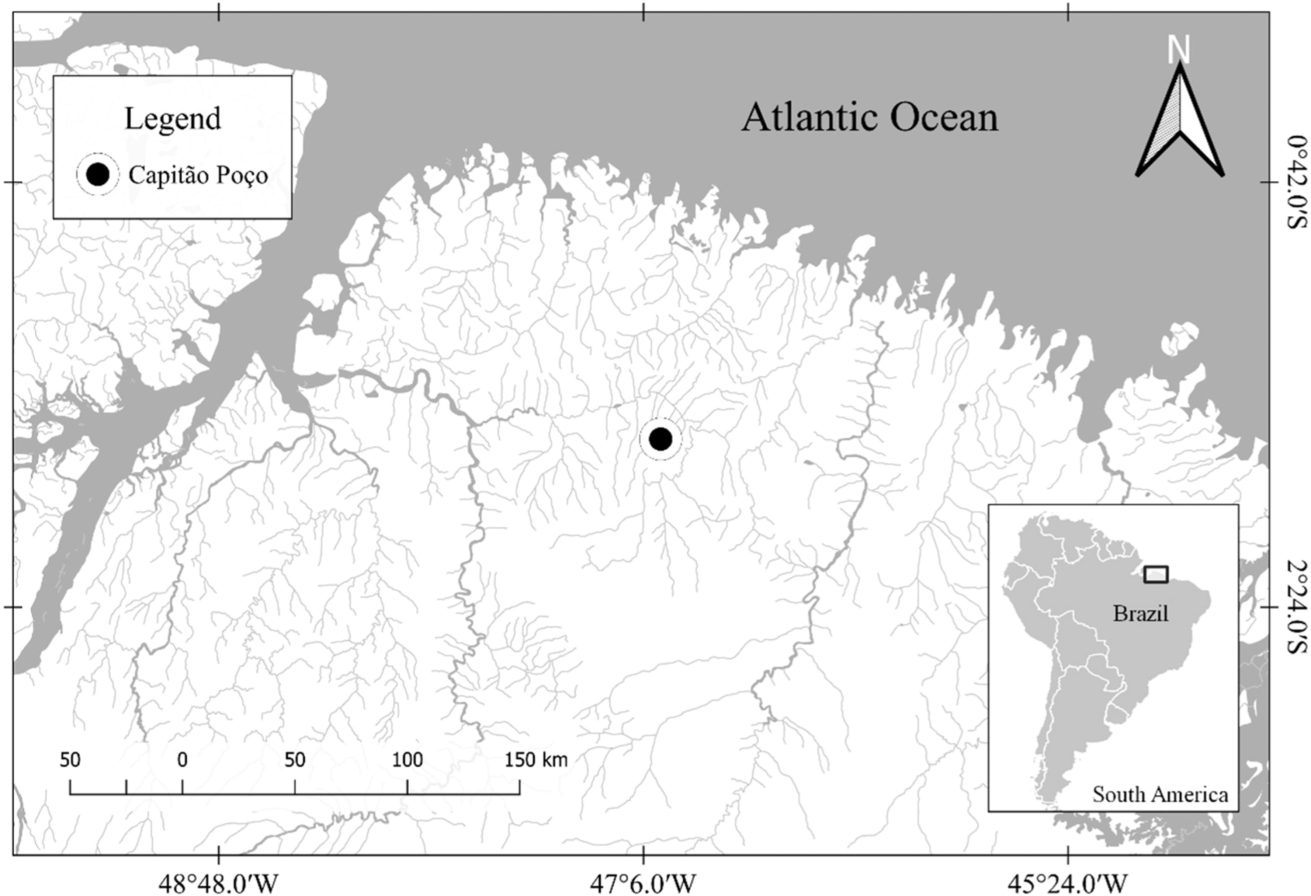
Map indicating sample collection locations of *P. unifilis* and *P. expansa* analyzed cytogenetically in this study.

### Chromosomal Preparation

Temporary lymphocyte cultures were carried out according to Moorhead et al.,^24^ with adaptations for the species under investigation. Approximately 0.5 mL of whole blood was inoculated in every 5 mL of RPMI-1640 medium (SIGMA). The samples were then placed in a CO_2_ incubator (5%) at 37 °C for 72 h for cell growth. 1 h before the end of the 72 h, 0.1 mL of colchicine (concentration 1:40) was added to the medium. After the end of cultivation, 5 mL of 0.075 M hypotonic solution (KCl), previously heated to 37 °C, was added for 20 min. Then, 1 mL of Carnoy’s fixative 4:1 (ratio 4 mL of methanol + 1 mL of acetic acid) was added. After centrifuging at 1000 rpm for 5 min, the supernatant was removed and 5 mL of Carnoy’s fixative was added so that the material could be stored at −20 °C for 3 days.

### DNA Extraction and PCR

The DNA extraction technique followed the method previously described by Sambrook et al.^25^ with a few modifications, using muscle tissue from the PEX and PUN species. The sequences of the genes encoding histones H2A, H2B, and H4 were amplified by polymerase chain reaction (PCR) using the following primers: H2A-ChH2AF: 5′-ATGTCAGGSCGAGGMAAG-3′; ChH2AR: 5′-TGGATGTTRGGCAGKACAC-3′; H2B-ChH2BF: 5′- GCCTGAGCCAGCRAAATCYG-3′; ChH2BR: 5′-ACTTRGAGCTGGTGTACTTRG-3′; H4-ChH4F: 5′-GTCGTGGTAAAGGTGGYAARG-3′; ChH4R: 5′-GAATCCGTACAGAGTRCGRC-3′. The quantities and concentrations of each reagent were: 1 μL of genomic DNA (80 ng), 1 μL of forward primer (0.2 μM), 1 μL of reverse primer (0.2 μM), 1 μL of dNTPs (2 mM), 0.25 μL of Taq DNA Polymerase (Invitrogen) 1 U, 1 μL of MgCl_2_ (1.5 mM), 1 μL of reaction buffer 1× (200 mM Tris, pH 8.4, 500 mM KCL), and 18.75 μL of de água. The amplification program set was as follows: 1 cycle of 95 °C (4 min)/30 cycles of 95 °C (1 min), 60 °C (1 min) and 74 °C­(2 min)/1 cycle 74 °C (5 min)/hold 4 °C.

### Probe Production

The PCR products were quantified using an Epoch Microplate Spectrophotometer (BioTek Instruments) with Gen5 2.03.1 software, later labeled by Nick Translation with BioNick Labeling System (Invitrogen) for biotin labeling or DIG-Nick Translation Mix (Roche) for digoxigenin labeling.

### Fluorescence In Situ Hybridization (FISH)

FISH procedure was carried out as described by Pinkel et al.^26^ The hybridization solution contained 2 μL of probe, formamide (50%), 2× SSC, and dextran sulfate and was denatured at 70 °C. The chromosomal DNA was denatured in 70% formamide at a temperature of 65 °C. Hybridization took place at 37 °C for 48 h. The probes were detected using avidin-CY3 or antidigoxigenin conjugated to FITC. DAPI combined with Vectashield antifade mounting medium was used to counterstain the chromosomes.

### Immunolocalization of Methylated DNA

The immunolocalization test for methylated DNA in chromosomes was carried out according to the protocol described by Rens et al.,^27^ with adaptations for the species investigated. To dehydrate the chromosomes, an alcohol series (70, 85, and 100%) was carried out for 5 min each. The chromosomal DNA was denatured in 2 M hydrochloric acid solution for 7 min, and then the slides were washed in 0.1 M sodium borate (ph = 8.4) for 1 min. Subsequently, the slides were washed in PBS 1× for 3 min, and again, the chromosomes were dehydrated in a series of alcohol solutions (70, 85, and 100%) for 5 min each and rehydrated in PBS 1× for 3 min. The primary antibodies 5-methylcytosine (5mC, Abcam, cat. Ab-124936) and 5-hydroxymethylcytosine (5hmC, Active Motif, cat. 39770) (1:600 in 1% BSA or PBT) were applied to the slides and kept for 12 h in a refrigerator (4 °C). After this incubation period, the slides were incubated in PBT 3 times for 10 min each. Secondary antibodies mouse polyclonal anti-5mC-TRITC and rabbit polyclonal anti-5hmC-FITC were added (1:300 BSA1%). The slides were covered with coverslips and kept for 1 h at room temperature. The slides were washed in PBT 3 times for 10 min each, and 7 ul of DAPI solution was added with Antifade Vectashield H-100 (Vector) and analyzed on a Zeiss epifluorescence microscope with AxioVision rel 4.6 software and Axiocam camera.

## Results

### Cytogenetic Analysis

The PEX and PUN species have a diploid number of 2*n* = 28 (Figure 2). Both species showed variation in fundamental number (NF): PEX has NF = 50, while PUN has NF = 46. Fluorescence *in situ* hybridization (FISH) showed clusters in the histone H2A genes located in the pericentromeric region in PUN in pairs 1, 2, 5, 6, 7, 9, 10, and 11 (Figure 3a). In PEX (Figure 3d), these genes were mapped in pairs 1, 2, 3, 4, 5, 6, 8, 9, 10, 11, 13, and 14, as well as a double marking in pair 3.

**Figure 2:**
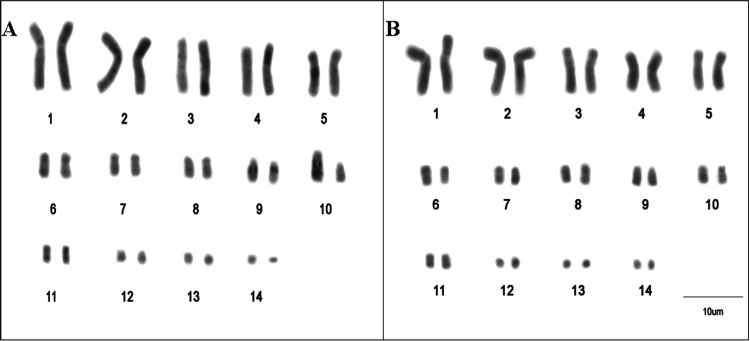
Karyotypes of *P. unifilis* (A) and *P. expansa* (B).

**Figure 3:**
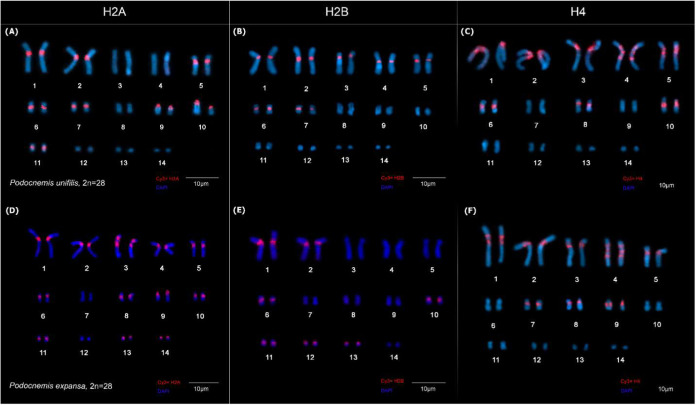
Fluorescent *in situ* hybridization (FISH) with a histone gene probe. The karyotypes of *P. unifilis* (A–C) and *Podocnemis expansa* (D–F) show cluster distribution of the histone genes H2A, H2B, and H4 (in red).

The histone H2B gene probe also showed clusters in the pericentromeric regions in pairs 1, 2, 3, 4, 5, 6, and 7 in PUN (Figure 3b) and in chromosome pairs 1, 2, 6, 10, 11, 12, and 13 in PEX (Figure 3e). Similarly, in PUN, these gene were observed in pairs 1, 2, 3, 4, 5, 7, 8, and 9 (Figure 3c), with pair 5 showing double marking and pair 4 showing triple marking similar to that observed in PEX; in PEX, the sequence of the histone H4 gene was observed organized in clusters in pairs 1, 2, 3, 4, 5, 6, 8, and 10, where in pair 1 it showed double marking, while in pair 4 it showed three markings (Figure 3f)

### DNA Methylation Analysis

Immunolocalization of methylated DNA markers in PUN and PEX showed that highly methylated regions (rich in 5mC) are almost homogeneously distributed along several pairs of karyotypes in both species studied. In relation to 5hmC, in PEX (Figure 4a), the distribution of this epigenetic mark was dispersed throughout the chromosome, in pairs 1, 2, 3, 4, 6, and 7. The results of immunolocalization with anti-5mC antibody in PEX (Figure 4b) showed 5mc-rich regions along the chromosome arms and in distal regions in pairs 4, 5, 6, 9, 12, 14, and 15, and concentrated in the pericentromeric region of pairs 2, 3, 4, 5,6, 7, 8, 9, 10, 12, and 13.

**Figure 4:**
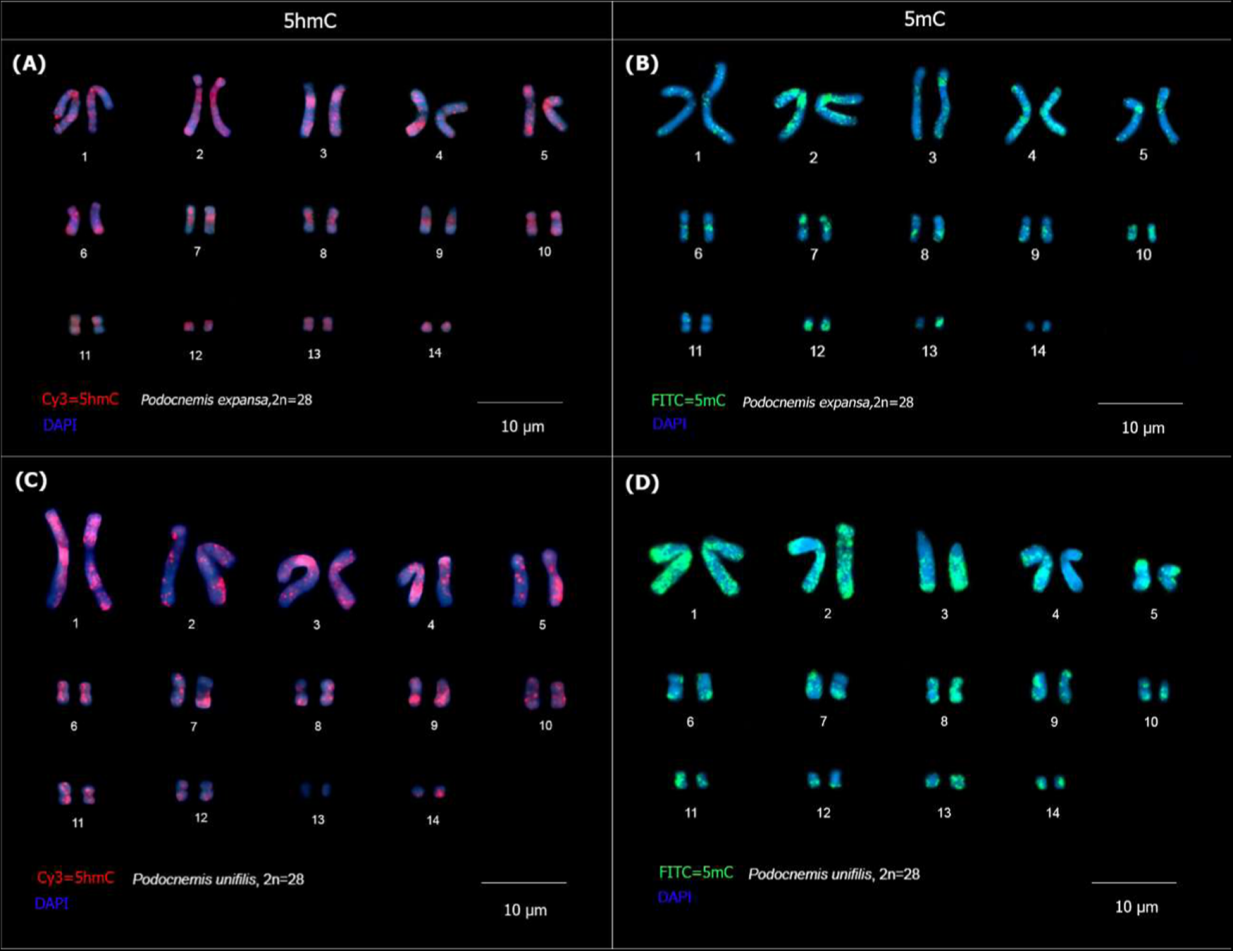
Immunolocalization of 5mC and 5hmC DNA in *P. expansa* (A, C) and *Podocnemis unifilis* (B, D). Arrangement of DNA markers methylated with anti-5hmC (red) and anti-5mC (in green) antibodies. The acronyms PEX and PUN refer to *P. expansa* and *P. unifilis*, respectively.

In PUN, it was possible to observe a dispersed distribution of 5hmC in almost all homologues, along the chromosome arms, except pair 13 (Figure 4c). The 5mC markings are dispersed along the chromosome arms of pairs 1, 2, 3, 4, 5, 7, 9, 13, and 14, with clusters in distal regions in pairs 6, 8, 10, and 12 (Figure 4d).

## Discussion

Current literature shows that DNA-hist chromosomal mapping has been investigated mainly in invertebrates and fish.^8,28,29^ Figure 5 shows an idiogram compiling the physical location of these genes recorded in PEX and PUN previously^12^ and from the present study. Note the occurrence of multiple clusters of different DNA-hist in the pericentromeric region in several pairs of the karyotype of both terrapins. Comparing the findings of the present study and the C-banding patterns in PUN and PEX described by Noronha et al.,^13^ it is inferred that the DNA-hist localization in these turtles is located within or adjacent to heterochromatin. Cavalcante et al.^12^ also reported similar results in relation to DNA-hist clusters H1 and H3 in same species. In some pairs, the five histone genes were present in the same chromosomal portion, as observed in pair 1 of PEX and PUN; similar results have already been observed in insects^30^ and bivalves.^31^ In pair 1 of PEX and PUN, the histone sequences H2A, H2B, and H4 coincide in their location with the distribution of H1 and H3, previously described in the same chromosomal locus, corroborating the idea that this is a region of conservation synteny in the Podocnemidae family.^12^ The clustering of the five histone genes in the same locus may contribute to their coordinated expression and facilitate the rapid activation of the expression of these multigenes during the S-phase of interphase.^32^

**Figure 5:**
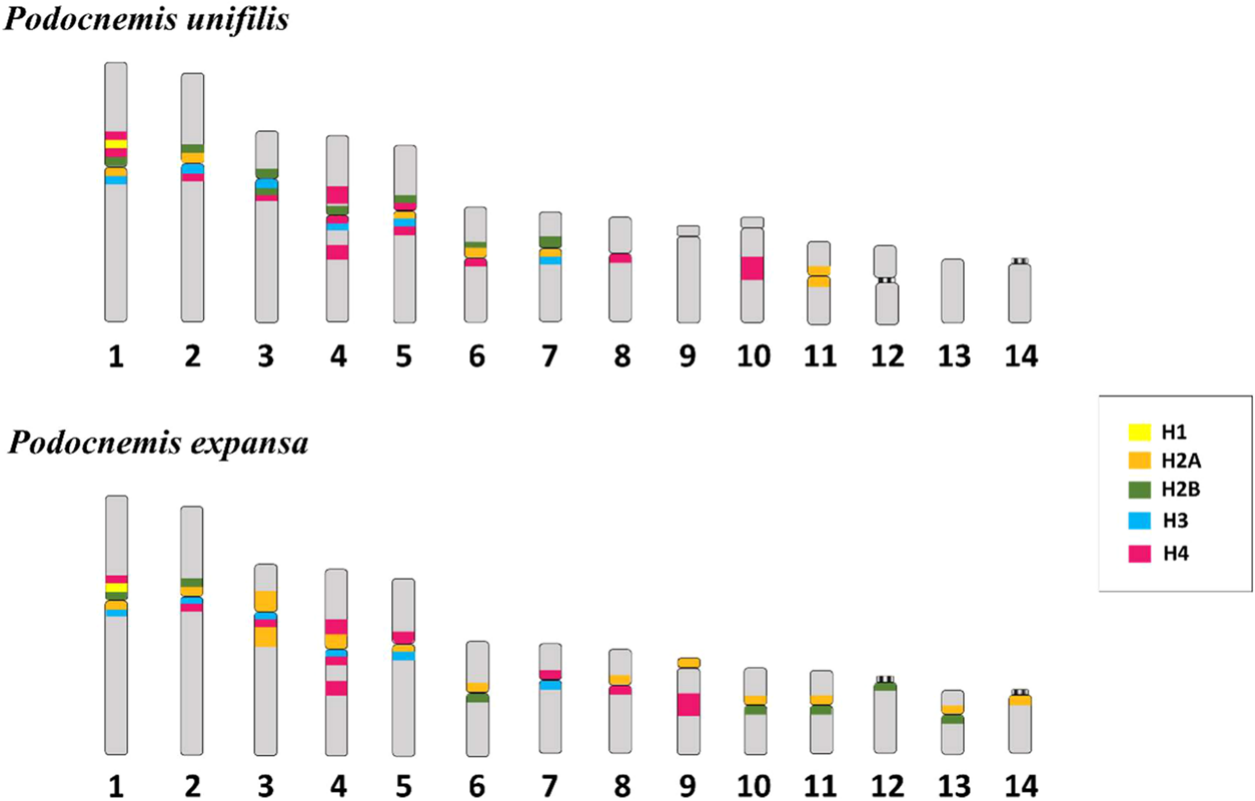
Ideogram illustrating the locations of the histone sequences investigated in this paper (H2A, H2B, and H4) in comparison with the histone sequences H1 and H3 from the previous study by Cavalcante et al.^12^; the acronyms PUN and PEX refer to *P. unifilis* and *P. expansa*, respectively.

The extensive dispersion of clusters of DNA-hist sequences observed in PUN and PEX in the present study may be the result of the transport of these sequences to other pericentromeric regions by ectopic recombination between satellite DNAs or transposable elements (TEs);^33^ the latter tend to be kept in heterochromatic regions due to the low recombination rate and functional genes in this part of the genome.^34^ Interestingly, the presence of mobile elements inserted into DNA-hist sequences has previously been reported in several vertebrates, such as fish,^6^ mammals,^7^ and the turtle *Rhinoclemmys punctularia*, whose histone H3 gene carries a partial Gypsy retrotransposon sequence;^14^ the physical proximity between karyotype pairs necessary for this process to occur can be obtained during the formation of the bouquet configuration at the beginning of meiosis, similar to that proposed for the histone H3 gene dispersal model in bivalves by García-Souto et al.^35^ Alternatively, DNA-hist dispersal in both *Podocnemis* species may be the result of an integration of extrachromosomal circular DNA, which can be generated by genomic rearrangements, duplications, or transposable element activities, playing an important role in biological processes such as gene expression and evolution, as observed in other multigenic families.^36^

Heterochromatin in reptiles features satellite DNAs, transposable elements, and genes, which perform different functions, such as regulating gene expression and chromatin formation.^37^ These sites rich in repetitive DNA can often constitute hostspots for chromosomal breaks and reorganizations, generating unconventional DNA secondary structures of high instability and interfering with processes such as replication, DNA repair, and transcription.^38^ Chromosomal rearrangements can alter the positions of multigenic families along the karyotype. In aphids, for example, subterminal clusters of histone H3 began to be located in interstitial regions of a compound chromosome after tandem fusion.^39^ In relation to PEX, additional histone H2A gene clusters were observed in the euchromatin of pair 3. We propose that a pericentric inversion-type rearrangement may have occurred by transferring part of the H2A sequences from the pericentromere to the interstitial region on the q-arm of this pair of homologues (Figure 6). A similar proposal was used to explain the distribution of 5S rDNA in the gymnotiform *Apteronotus albifrons* ^40^ and in *Drosophila melanogaster*.^41^ In contrast, the same reasoning can be applied to elucidate the markings of the histone H4 gene in the p and q arms of pair 4 of both species in heterochromatic regions but in this case through two independent inversion events. Previous studies have demonstrated the occurrence of pericentric inversions in pairs 13 ^42^ and 10^13^ of PUN and PEX, indicating that these alterations are frequent in their genomes and that they may possibly be the main mechanisms of karyotypic diversification in the *Podocnemis* genus.

**Figure 6:**
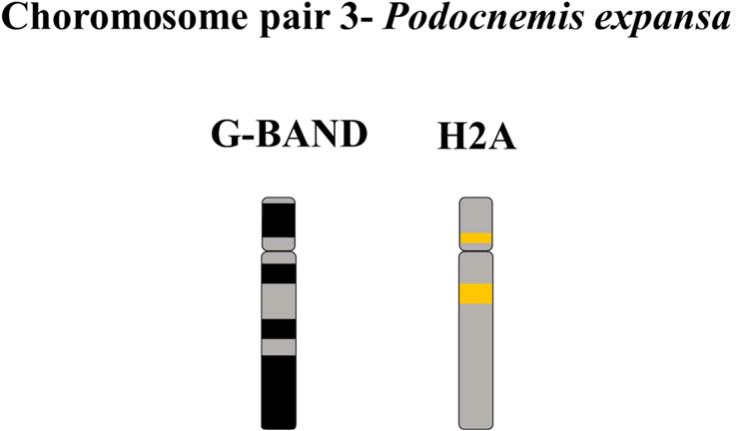
Ideogram of the common chromosomal region of chromosomal pair 3 of *P. expansa*. G bands described previously by Noronha et al.^13^

Immunolocalization of 5-methylcytosine in PUN and PEX showed methylated regions along the lengths of the chromosome arms in both species. In this context, it is possible to propose that the global DNA methylation patterns visualized in this study are important for genomic maintenance as they perform, among other functions, the repression of transposable elements and regulation of gene expression.^43^ Hypermethylated loci were recorded, by immunolocalization, coinciding with the chromosomal distribution of the transposon Mariner and LINE in the fish *Astyanax scabripinnis*, suggesting a mechanism of inactivation of these TEs by methylation.^44^ In *Podocnemis*, a similar inference can be made, since the distribution of 5mC coincides in many chromosomal regions with loci occupied by the transposable elements *Mariner* ^45^ and *Rex*6 ^13^ located on the p and q arms of several pairs of the karyotype of both turtles. In this study, we also evaluated, for the first time in reptile chromosomes, the localization of 5hmC, which originates from the conversion of 5mC, by enzymes of the ten–eleven-translocation (TET) oxygenase family. This conversion can lead to DNA demethylation displaying an open chromatin configuration as well as activation of gene expression. In fact, studies have shown that 5hmc is an epigenetic mark characteristic of gene-rich genomic regions with high transcriptional activity.^46,47^ This explains the distribution of 5hmc in euchromatic regions of the *Podocnemis* chromosomes.

In addition, the results showed that, especially in PEX, there is greater accumulation of 5mC in the pericentromeric regions of chromosomes. DNA methylation occurs most frequently in CpG islands, which contain a high concentration of cytosine and guanine, although there is evidence that methylation can occur in other ways.^48^ Considering this information, the high concentration of GC bases in pericentromeric regions of the PUN and PEX chromosomes demonstrated previously through banding by chromomycin A3 ^13^ may partly explain possible hypermethylation observed in this portion of the genome, specifically in pair 1 of PUN and PEX (Figure 7), thus suggesting that regions of colocalization of histone genes and accumulation of 5mC may be associated with the silencing of these genes, interfering with the gene expression of histone genes. However, more analysis needs to be carried out to confirm this hypothesis, so these are preliminary results for understanding the karyotypic diversification of chelonians.

**Figure 7:**
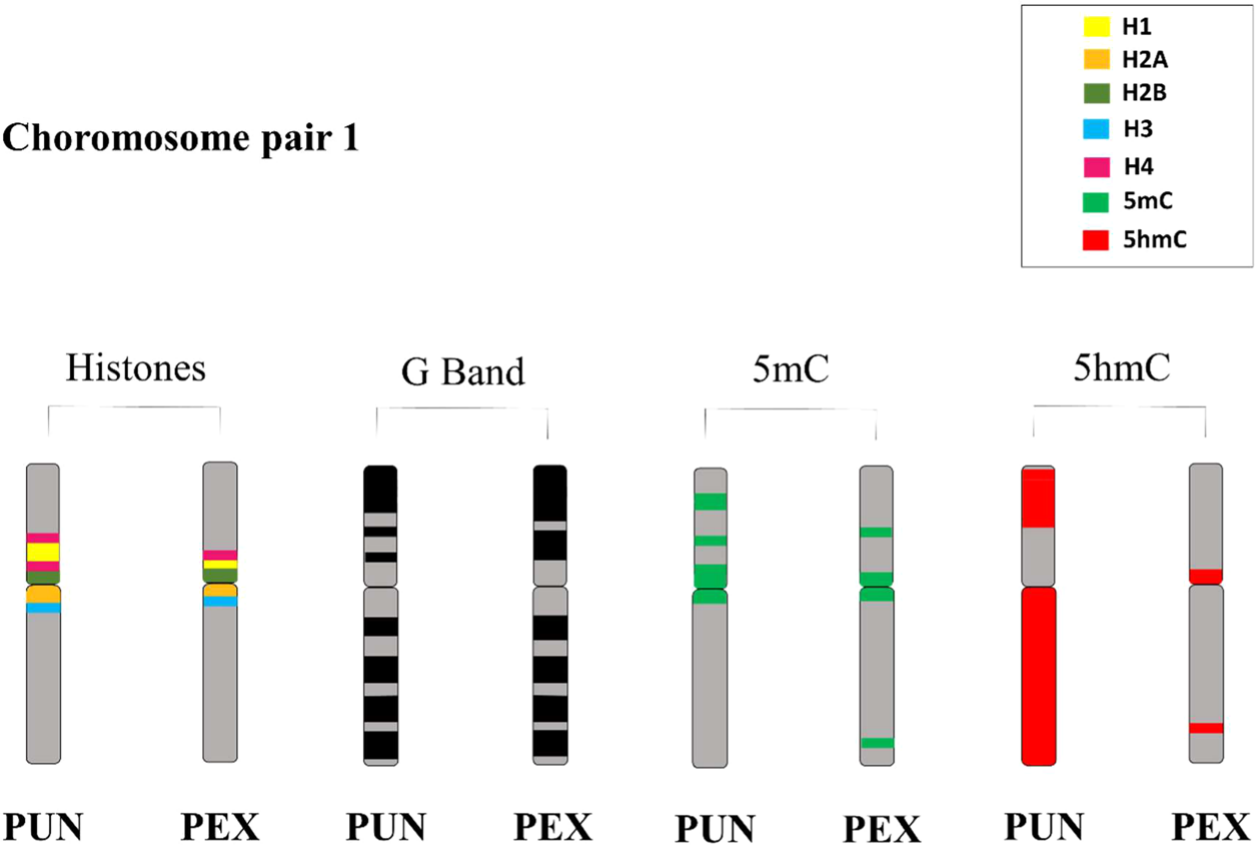
Ideogram of the common chromosomal region of the first chromosome pair of *P. unifilis* and *P. expansa*. G bands described previously by Noronha et al.^13^

Regarding the chromosome ends of PUN and PEX, the abundance of 5mC in these regions is not an exclusive feature of these species, as previous studies have shown similar results in mammals,^49^ lizards,^50^ and some birds.^51^ It is suggested that this methylation pattern seen in the distal portions of chromosomes refers to the subtelomeric region, considering that the repetitive sequences that constitute it have a high GC content in eukaryotes and the fact that the telomeric repeat of vertebrates—TTAGGG—does not have the GC dinucleotide necessary for the formation of 5mC.^52^ Methylation in pericentromeres and subtelomeres is of great importance in the genome as a homeostatic benefit and for telomere stability.

The relationship between 5mC and its participation in the modulation of DNA-hist expression has been little studied in vertebrates. Although the immunofluorescence and FISH techniques used in this study show us a macrostructural pattern of the karyotype, comparative analysis of the physical distribution of DNA-hist and methylated regions in PUN and PEX allows us to hypothesize that most of the histone gene clusters present in the pericentromeric region of the heterochromatin of these turtles may be inactivated by interference in their expression through the methylation of their promoters and by the recruitment of proteins that cause DNA compaction.^53^ The presence of at least two methylated CpG islands in cis-regulatory elements of the histone H4 gene have been shown to be sufficient to prevent or drastically reduce the interaction between its promoter and transcription factors, inhibiting its gene expression.^54^ Other clusters located in euchromatin, such as the histone H4 mark on the q-arm of pair 4 in PEX, coinciding with a band rich in 5mC (Figure 5), may also be silenced by the same mechanisms. This hypothesis can also be corroborated by the fact that the canonical histone genes that make up nucleosomes (the targets of this study) have replication-dependent expression, i.e., their transcriptional activity is limited to S-phase.^4^ Therefore, we suggest that in the PUN and PEX karyotypes analyzed in the present study, hypermethylated chromosomal regions may contribute to the inactivation of DNA-hist during the cell cycle. Additionally, transcriptional silencing of these genes in these turtles may be the result of the effects of DNA methylation and other diverse epigenetic processes that act at transcriptional, translational, and post-translational levels.^55^
